# Glucagon-like peptide-1 receptor agonist Liraglutide has anabolic bone effects in ovariectomized rats without diabetes.

**DOI:** 10.1371/journal.pone.0132744

**Published:** 2015-07-15

**Authors:** Nan Lu, Hanxiao Sun, JingJia Yu, Xiaojing Wang, Dongmei Liu, Lin Zhao, Lihao Sun, Hongyan Zhao, Bei Tao, Jianmin Liu

**Affiliations:** Department of Endocrine and Metabolic Diseases, Rui-jin Hospital, Shanghai Jiao-tong University School of Medicine, Shanghai Institute of Endocrine and Metabolic Diseases, Shanghai Clinical Center for Endocrine and Metabolic Diseases, Shanghai 200025, China; University of Lancaster, UNITED KINGDOM

## Abstract

Recently, a number of studies have demonstrated the potential beneficial role for novel anti-diabetic GLP-1 receptor agonists (GLP-1RAs) in the skeleton metabolism in diabetic rodents and patients. In this study, we evaluated the impacts of the synthetic GLP-1RA Liraglutide on bone mass and quality in osteoporotic rats induced by ovariectomy (OVX) but without diabetes, as well as its effect on the adipogenic and osteoblastogenic differentiation of bone marrow stromal cells (BMSCs). Three months after sham surgery or bilateral OVX, eighteen 5-month old female Wistar rats were randomly divided into three groups to receive the following treatments for 2 months: (1) Sham + normal saline; (2) OVX + normal saline; and (3) OVX + Liraglutide (0.6 mg/day). As revealed by micro-CT analysis, Liraglutide improved trabecular volume, thickness and number, increased BMD, and reduced trabecular spacing in the femurs in OVX rats; similar results were observed in the lumbar vertebrae of OVX rats treated with Liraglutide. Following *in vitro* treatment of rat and human BMSCs with 10 nM Liraglutide, there was a significant increase in the mRNA expression of osteoblast-specific transcriptional factor Runx2 and the osteoblast markers alkaline phosphatase (ALP) and collagen α1 (Col-1), but a significant decrease in peroxisome proliferator-activated receptor γ (PPARγ). In conclusion, our results indicate that the anti-diabetic drug Liraglutide can exert a bone protective effect even in non-diabetic osteoporotic OVX rats. This protective effect is likely attributable to the impact of Liraglutide on the lineage fate determination of BMSCs.

## Introduction

Glucagon-like peptide-1 (GLP-1) receptor agonists (GLP-1RAs) are a new class of anti-diabetic medications that mimic the effects of incretin hormones [1]. GLP-1, as an incretin hormone which is synthesized and secreted from gut L cells in response to food intake, can stimulate insulin release by pancreatic β-cells in a glucose-dependent manner and suppress glucagon secretion from α-cells[2]. In addition to its glucose-lowering effect, GLP-1 also delays gastric emptying and inhibits appetite, possibly by affecting vagal afferent fibers and the hypothalamus, eventually leading to weight loss [3, 4]. These favorable actions of GLP-1 on glucose homeostasis are mediated through GLP-1 receptors. However, native GLP-1 is rapidly degraded in circulation by the enzyme dipeptidyl peptidase IV (DPP-IV) [5]. Liraglutide, a synthetic GLP-1RA that share 97% homology with the structure of human GLP-1, is resistant to DPP-IV inactivation and possesses a much longer circulating half-life, thereby making it a novel anti-diabetic drug suitable for once-daily injection[1].

In addition to their usefulness in treating diabetes and obesity[6], the presence of GLP-1 receptors in various tissues other than pancreatic β -cells and α -cells (i.e., brain and heart) has aroused a great deal of interest in exploring the cardiovascular, neuroprotective, renal protective and other extra-pancreatic benefits of GLP-1RAs [7–10]. *In vitro* studies showed that osteoblastic cells express functional receptors for GLP-1 [11]; thus, bone might also be a potential target organ for GLP-1RAs. Indeed, continuous subcutaneous infusion of GLP-1 or extendin-4 (a naturally occurring analogue of GLP-1) for 3 days in both insulin-resistant and type 2 diabetic rat models normalized their impaired trabecular architecture and promoted bone formation via an insulin-independent mechanism [12, 13]. GLP-1 administration can also restore bone mineral densities (BMDs) in rats with hyperlipidemia-induced bone loss [14]. These findings highlighted the potential use of GLP-1RAs in combating diabetes-related bone defects.

Bone loss can also arise from estrogen deficiency. Therefore, an interesting question remains concerning whether GLP-1RAs can effectively treat osteoporosis in animal models without diabetes. Exendin-4 was recently shown to exhibit an osteogenic effect in ovariectomized (OVX) rats [15]; however, whether daily Liraglutide injections can induce similar anabolic bone effects in osteoporotic rodents without diabetes is not clear.

GLP-1 receptors are also expressed on bone marrow stromal cells (BMSCs) [16]. Multipotent BMSCs can differentiate into adipocytes, osteoblasts, chondrocytes or myocytes under the control of several transcription factors [17, 18]. Because there is a mutually exclusive relationship between the differentiation of adipocytes and osteoblasts from their common progenitor cells, it is necessary to investigate whether a GLP-1RA such as Liraglutide can affect the lineage fate determination of BMSCs.

Therefore, the current study was performed in OVX rats without diabetes to ascertain that the bone effects of this anti-diabetic drug could also be demonstrated in osteoporotic rats in a manner that was independent of its blood glucose-lowering effects. Additionally, we investigated the regulatory actions of Liraglutide on osteoblastogenesis and adipogenesis in rat and human BMSCs.

## Materials and Methods

### Animals and treatment

Eighteen 6-week-old female Wistar rats were used in this study. The rats were bred in Shanghai Slac Laboratory (Slac Laboratory Animal Co. Ltd, Shanghai, China) and raised in Shanghai Jiao-Tong University. The animals were housed individually in cages in an air-conditioned environment with stable temperature (22°C±2°C), humidity (50% to 70%) and a 12‐hour light‐dark cycle (lights on at 8:00 A.M.), and provided access to a standard chow diet and water *ad libitum*. The use of laboratory animals in this study was approved by the Animal Care Committee of the Shanghai Jiao-Tong University and performed under accepted ethical guidelines.

After a 14 day acclimatization period, the rats (8-weeks-old) were anesthetized via an intraperitoneal injection of 5% chloral hydrate (8 ml/kg) and underwent either a sham surgery or bilateral ovariectomy. Three months after the surgery, the rats (5 months of age) were randomly divided into three groups to receive the following treatment for 2 months: (1) Sham + normal saline; (2) OVX + normal saline; and (3) OVX + Liraglutide. Rats in the OVX+ Liraglutide group were given once daily s.c. injections of Liraglutide (Novo Nordisk A/S, Denmark) at a dose of 0.6 mg/day at 8 A.M. Normal saline was used as the vehicle and was subcutaneously injected into the control rats daily.

The body weight and fed state blood glucose levels were monitored weekly. Prior to euthanasia, an intra-peritoneal glucose tolerance test (IPGTT) was performed as follows: after overnight fasting, the animals were given an injection of glucose solution (2 g/kg body weight). Blood samples was collected from the caudal vein immediately prior to glucose administration and then 15 min, 30 min, 60 min, and 120 min after for the measurement of glucose levels using a glucose reagent strip and glucometer (One-Touch Ultra, Lifescan). At the end of the experiment, the rats were euthanized by ether inhalation. Afterwards, the femurs and the fifth lumbar vertebrae were harvested and carefully stripped of soft tissue attachments at 4°C to avoid any damage to the bones. The right femurs and the lumbar vertebrae were stored in 4% paraformaldehyde in 0.1 M sodium phosphate buffer (PBS, pH 7.4) for the micro-CT evaluation.

### Micro-CT evaluation

Bone samples stored in 4% paraformaldehyde for 24 hours were dehydrated with 70% ethyl alcohol for 48 hours, then positioned in a custom jig with 40% ethyl alcohol and monitored using a micro-CT scanner at a 14 μm resolution (GE Explore Locus SP Specimen Scanner, GE Care Co., London). The mineralized tissues were differentially segmented using a global thresholding procedure [19]. Briefly, for the trabecular bone analysis the femurs were scanned from 1.0 mm below the lower end of the growth plate and at the mid-portion of the lumbar vertebrae. For the cortical bone analysis, the mid-portion of the femurs was scanned. The following morphometric parameters from the binarized volumes of interest were analyzed via the Explore MicroView version 2.2 (GE Healthcare): bone volume ratio (BV/TV), trabecular number (Tb.N), trabecular thickness (Tb.Th), trabecular separation (Tb.Sp) and cortical thickness.

### Cell culture

The effects of Liraglutide on osteoblastogenic and adipogenic differentiation *in vitro* were determined by culturing rat and human BMSCs in different differentiation media with or without 10 nM Liraglutide.

Rat BMSCs were derived from 4-week-old male Wistar rats, and both adipogenesis and osteoblastogenesis were induced as previously described [20]. Primary human BMSCs were gifts from the Shanghai Key Laboratory of Orthopedic Implant. The human BMSCs were donated by patients with written informed consent, and this experiment was approved by the Independent Ethics Committee of Shanghai Ninth People’s Hospital affiliated with Shanghai Jiao Tong University School of Medicine (SJTUSM)[21]. For adipocyte differentiation, human BMSCs were initiated with an adipogenic induction medium (AIM): DMEM-low glucose media supplemented with 15% FBS, 10μM insulin, 1μM dexamethasone, 0.5 mM 3-isobutyl-1-methylxan-thine (IBMX), and 200μM indomethacin in the presence or absence of Liraglutide. Thereafter, the AIM was replaced with adipogenic maintenance medium (AMM) consisting of DMEM-low glucose, 15% FBS, and 10 μM insulin in the presence or absence of Liraglutide for 1 day, and then the cells were switched back to AIM. Alternate incubations with the two different media were repeated every 3 days [16]^.^ For osteoblastogenic differentiation, human BMSCs were cultured in DMEM-low glucose media supplemented with 15% FBS, 100 nM dexamethasone, 10 mM 2-glycerol phosphate and 0.05 mM ascorbic acid [22, 23].

Lipid droplets within the differentiated adipocytes from rat and human BMSCs were observed using a modified Oil Red O staining method [16] after 14 and 17 days of adipogenic differentiation, respectively. Briefly, the cells were washed twice with PBS, and then fixed for 5 min with 4% formaldehyde. Fixed cells were incubated with Oil Red O for 30 min at room temperature, followed by three washes with PBS. Oil Red O was prepared by diluting 60 mL of stock solution (0.5 g in 100 mL of isopropanol) in 40 mL of distilled water and filtered through coarse filter paper prior to use. After staining with the Oil Red O, the cells were washed and photographed. The dye retained by the cells was eluted by incubation with isopropanol for 15 minutes and quantified by absorbance measurements at 510 nm (Safire2, TECAN). One week after osteoblastogenic induction, alkaline phosphatase (ALP) staining was accomplished with the use of an ALP staining kit (Renbao, Shanghai, China)[24]. Alizarin red staining was performed for the visualization of calcium deposits [25] after 21 days of osteoblastogenesis. Cells were incubated with 2% alizarin red (pH 4.2) for 10 minutes, then subsequently washed with distilled water and photographed. Calcium deposits were extracted with 1 mL of 0.1 N sodium hydroxide, and the optical density was recorded at 548 nm (Safire2, TECAN).

### Real-time PCR

Total RNA was extracted from the cells described above with the TRIzol reagent (Invitrogen). Then, the extracted RNA was reversed-transcribed using the Reverse Transcription System (Promega, Madison, WI, USA). The PCR primer sequences are provided in Table 1. PCR amplification was performed using SYBR Premix Ex Taq (TAKARA, Japan) in a Light Cycler instrument (Roche). The thermal cycling conditions for the PCR reactions were 95°C for 30 seconds, followed by 40 cycles of 95°C for 10 seconds, 60°C for 20 seconds, and 95°C for 15 seconds and ending with 60°C for 15 seconds. All reactions were performed in triplicate. The mRNA expression levels were normalized to the endogenous control GAPDH. The relative expression levels of control and Liraglutide treated samples at different time points over day 0 (0d) control sample were calculated with the 2^-ΔΔCt^ method, where -ΔΔCt = ΔCt _treatment_ -ΔCt _0d control_.

**Table 1:** Primers sequences used in the study.

Gene	primer sequences
**Rat primers**	
GAPDH	CTCAACTACATGGTCTACATGT (Forward)
	CTTCCCATTCTCAGCCTTGACT (Reverse)
Runx2	ACTGGCGGTGCAACAAGA (Forward)
	TAGTTCTCATCATTCCCGGC (Reverse)
ALP	CCAAAAACTCAACACCAACG (Forward)
	TCCATCTCCAGCCGTGTCT (Reverse)
Col-Ⅰ	TGTCCCAACCCCCAAAAA (Forward)
	ATGACTTCTGCGTCTGGTGAT (Reverse)
PPARᵧ	TGACCCAATGGTTGCTGATT (Forward)
	GCCTGTTGTAGAGTTGGGTTT (Reverse)
**Human primers**	
GAPDH	GAAGTGAAGGTCGGAGTC (Forward)
	GAAGATGGTGATGGGATTTC (Reverse)
Runx2	CTACCACCCCGCTGTCTTC (Forward)
	CAGAGGTGGCAGTGTCATCA (Reverse)
ALP	CCCCGTGGCAACTCTATCTTT (Forward)
	GATGGCAGTGAAGGGCTTCTT (Reverse)
PPARᵧ	TTGACCCAGAAAGCGATTCC (Forward)
	AAAGTTGGTGGGCCAGAATG (Reverse)

### Statistics

Data are presented as the mean ± SEM. Statistical analyses were performed with SPSS software (version 18.0). Variables were tested for normality using the Kolmogorov-Smirnov Z statistic. Group comparisons were evaluated with the one-way ANOVA test. Homogeneity of variance was assessed using Levene's test (Tb.N, Tb.Th, cortical BMD and Tb.BMD of the femur and Tb.Th of the lumbar vertebrae had equal variance; BV/TV, Tb.Sp, and cortical.Th of the femur and BV/TV, Tb.N, Tb.Sp, and Tb.BMD of the lumbar vertebrae had heterogeneous variance). The LSD test was used when equal variance was assumed; Tamhane's T2 test was used when equal variance was not assumed. P < 0.05 was considered statistically significant.

## Results

### Liraglutide prevented bone loss induced by OVX

**Fig 1:**
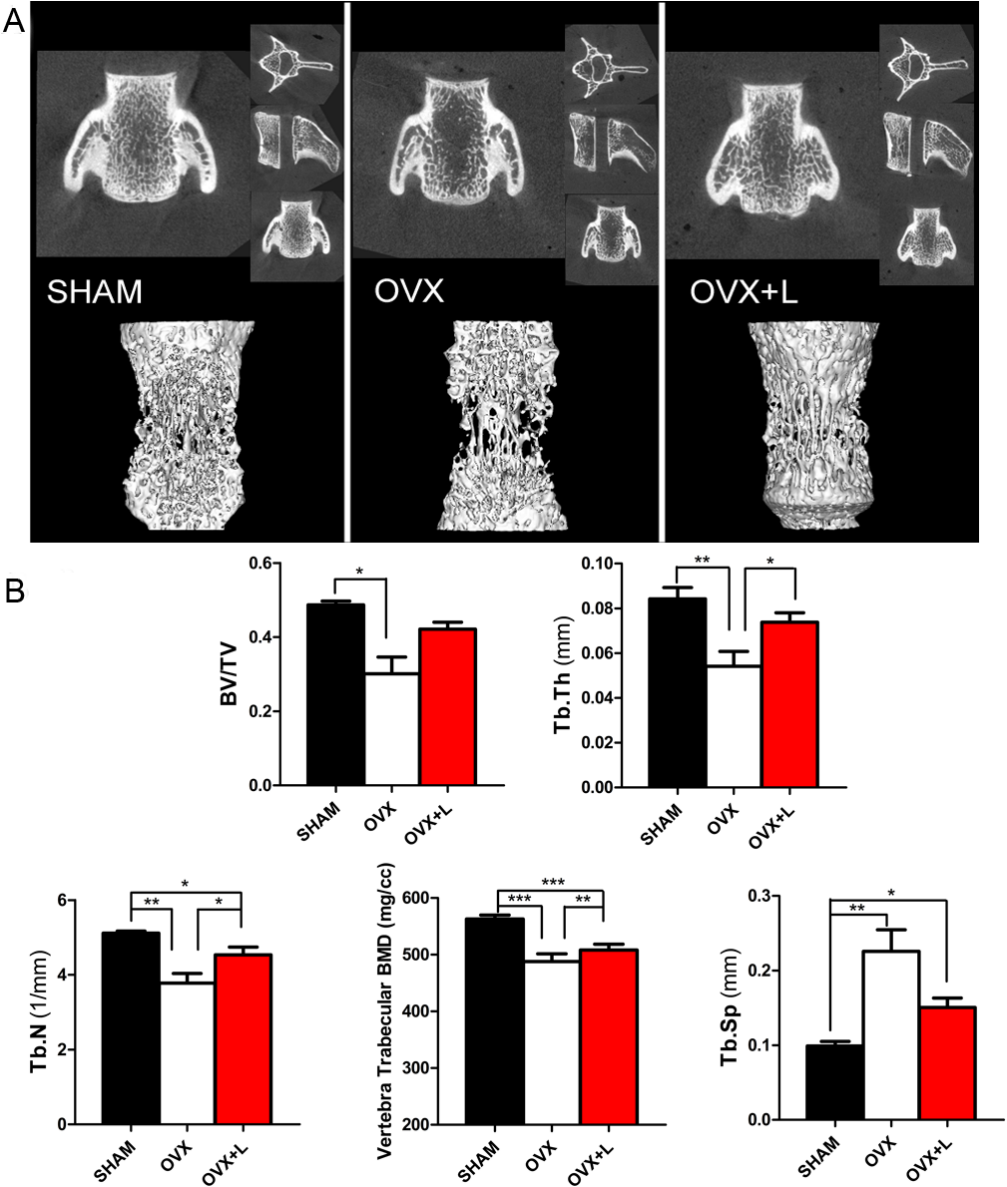
Effects of Liraglutide on the bone micro-architecture of the vertebra in OVX rats. (A) Representative micro-CT images of the 5th lumbar vertebra. The upper row presents the coronal view, axial view and sagittal view in the different groups, while the lower row provides a three-dimensional visualization of the trabecular micro-architecture. (B) The trabecular micro-architecture parameters of the 5th lumbar vertebra were shown as: BV/TV, Tb.Th, Tb.N, trabecular BMD, and Tb.Sp. Eight-week-old female Wistar rats were subjected to a sham ovariectomy (SHAM, black bar) or ovariectomy surgery (OVX); then, 3 months after surgery when the rats had established osteoporosis, vehicle (OVX, white bar) or Liraglutide (OVX+L, red bar) were administered for 8 weeks. Values are expressed as the mean±SE; n = 6 rats per group. *, P< 0.05; **, P< 0.01, ***, P<0.001.

**Fig 2:**
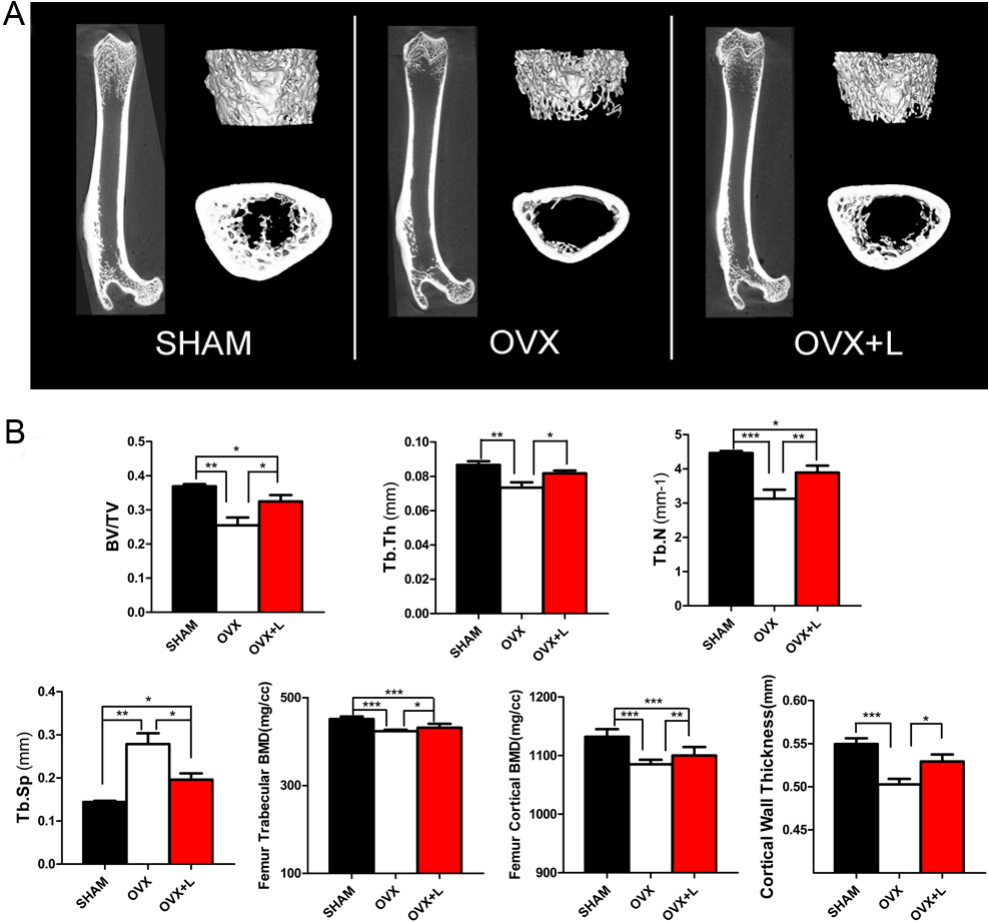
Effects of Liraglutide treatment on the bone micro-architecture of the femur in OVX rats. (A) Representative micro-CT images of the right femur. (B) The trabecular micro-architecture parameters of the distal end of the femur were shown as: BV/TV, Tb.Th, Tb.N, Tb.Sp, and trabecular BMD. The cortical parameters were also exhibited as the cortical BMD and cortical wall thickness. Eight-week-old female Wistar rats were subjected to a sham ovariectomy (SHAM, black bar) or ovariectomy surgery (OVX); then, 3 months after surgery when the rats had established osteoporosis, vehicle (OVX, white bar) or Liraglutide (OVX+L, red bar) were administered for 8 weeks. Values are expressed as the mean±SE; n = 6 rats per group.*, P<0.05; **, P<0.01; ***, P<0.001.

As shown in the different dimension views, the bone mass and bone cortical thickness were decreased and the trabecular micro-architecture was markedly impaired in OVX rats compared to sham-operated rats. These decreases were significantly improved by Liraglutide treatment (Figs 1A and 2A).

Liraglutide treatment of OVX rats induced a significant improvement in trabecular thickness by 36% (P = 0.023), trabecular number by 20% (P = 0.039), and trabecular BMD by 4% (P = 0.001) compared to their OVX littermates treated with vehicle in the fifth lumbar vertebra. An improvement in trabecular volume (BV/TV) and decrease in trabecular spacing were also observed, although the differences between groups did not reach statistical significance (Fig 1B). These anabolic effects of Liraglutide were also observed in the long bones. Analysis of the right femur using micro-CT displayed an increase in BV/TV by 27% (P = 0.049), cortical thickness by 5% (P = 0.020), cortical BMD by 1% (P = 0.008), trabecular thickness by 11% (P = 0.033), trabecular number by 24% (P = 0.002), and trabecular BMD by 2% (P = 0.015), and a decrease in trabecular spacing by 42% (P = 0.032) in the Liraglutide-treated OVX rats (Fig 2B). As shown in Fig 1B and Fig 2B, most bone indices in the OVX+Liraglutide group were still significantly worse than those of the SHAM rats.

### Liraglutide affected body weight and blood glucose levels in OVX rats

**Fig 3:**
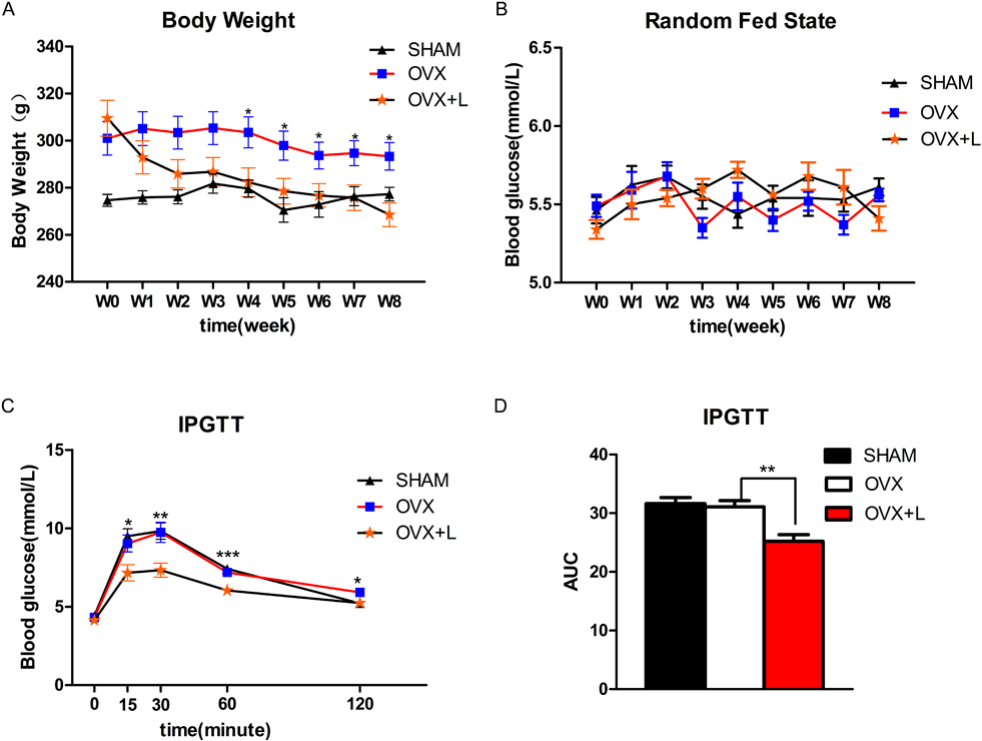
Effects of Liraglutide on body weight and blood glucose levels in OVX rats. The body weight (A) and random fed state blood glucose levels (B) were monitored weekly in vehicle-treated sham-operated Wistar rats (SHAM), vehicle-treated OVX rats (OVX) and Liraglutide-treated OVX rats (OVX+L) during 8 weeks of treatment with Liraglutide. Prior to euthanasia, an intraperitoneal glucose tolerance test (IPGTT) was performed. The glucose level and the area under curve (AUC) of the glucose tolerance in IPGTT (C, D) were also assessed. Values are expressed as the mean±SE; n = 6 rats per group.*, P<0.05; **, P< 0.01, ***, P<0.001; OVX vs. OVX+L.

As shown in Fig 3A, the OVX rats were heavier than their sham littermates. Daily Liraglutide administration for 8 weeks induced significant weight loss beginning at the 4th week. After 8 weeks of treatment, the body weight of OVX+Liraglutide rats were significantly lower than the OVX+ normal saline group, with an 8.4% difference between groups (268.5±17.1 g vs 293.3±11 g;P = 0.014).

The OVX and sham-operated rats had identical fed state blood glucose levels and similar glucose tolerance revealed by IPGTT (Fig 3B–3D). Interestingly, Liraglutide improved glucose tolerance in the OVX rats (Fig 3C and 3D).

### Liraglutide inhibited adipogenesis and enhanced osteoblastogenesis in rat and human BMSCs

**Fig 4:**
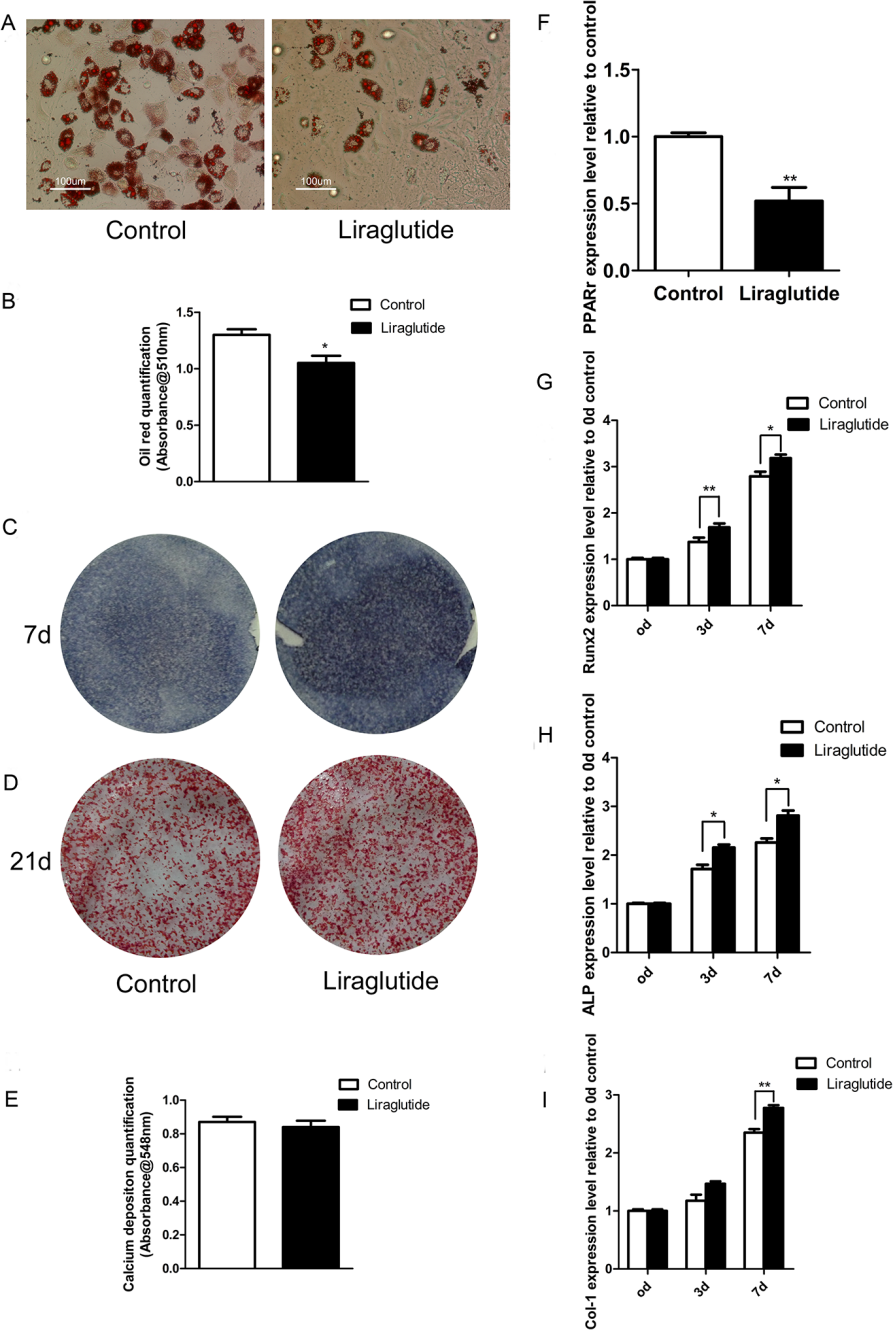
Effects of Liraglutide on adipogenesis and osteoblastogenesis of rat BMSCs. Primary rat BMSCs were harvested from the femur and tibia bone marrow from 4-week-old male Wistar rats. After 3 passages, the rat BMSCs were induced to differentiate into adipocytes or osteoblasts in the presence or absence of 10 nM Liraglutide. (A) Following 14 days of treatment after adipogenic inducement, the cultures were fixed and stained with Oil red O, then visualized and photographed. (B) The amount of Oil Red O in the cells was quantified. (C) ALP staining, which indicates the early stage of osteoblastogenesis, and (D) Alizarin red staining, which represents the mineralization levels of later stages of osteoblastogenesis, was performed at the indicated time-points. (E) Alizarin red dye was analyzed. (F) PPARγ, a crucial gene promoting adipogenesis, was quantified using real-time PCR after 14 days of adipogenesis. (G. H. I) The expression patterns of osteoblastogenic differentiation markers (Runx2, ALP, Col-1) of rat BMSCs in response to 10 nM Liraglutide were assessed using real-time PCR. Relative expression levels of each gene were calculated using the 2-ΔΔCt method. The expression of GAPDH mRNA was used for reference. Values are expressed as the mean ±SE. *, P<0.05; **, P<0.01; ***, P<0.001.

We hypothesized that the beneficial bone effects of Liraglutide were possibly attributable to its impacts on adipogenesis and osteoblastogenesis of rat BMSCs. As shown in Fig 4A, Liraglutide significantly inhibited the formation of adipocytes. Oil red O staining demonstrated the presence of numerous intracellular lipid droplets in the control, while significantly smaller or fewer lipid droplets were observed in the Liraglutide-treated cells. The Oil Red O in cells was quantified after decoloring, and a significant decrease was observed in the Liraglutide treated group (Fig 4B). These effects were supported by the real-time PCR analysis, which showed that the expression of the adipocyte-specific transcription factor peroxisome proliferator-activated receptor γ (PPARγ) was significantly down-regulated following Liraglutide treatment (Fig 4F).

Conversely, there was an obvious increase in ALP staining when Liraglutide was added for 7 days to cultures of rat BMSCs undergoing induced osteoblastogenic differentiation (Fig 4C). Real-time PCR showed that Liraglutide fully promoted an increase in the expression of Runx2, a transcription factor that is essential for osteoblast differentiation. These phenotypic changes were also accompanied by the elevated expression of key markers for osteoblastogenesis, such as ALP and Col-1 (Fig 4G, 4H and 4I).

Alizarin staining was performed after 21 days of osteoblastogenic culture to assess mineral deposition as a late marker of mature osteoblast function; however, we failed to observe clearly enhanced alizarin deposition following Liraglutide treatment (Fig 4D and 4E).

**Fig 5:**
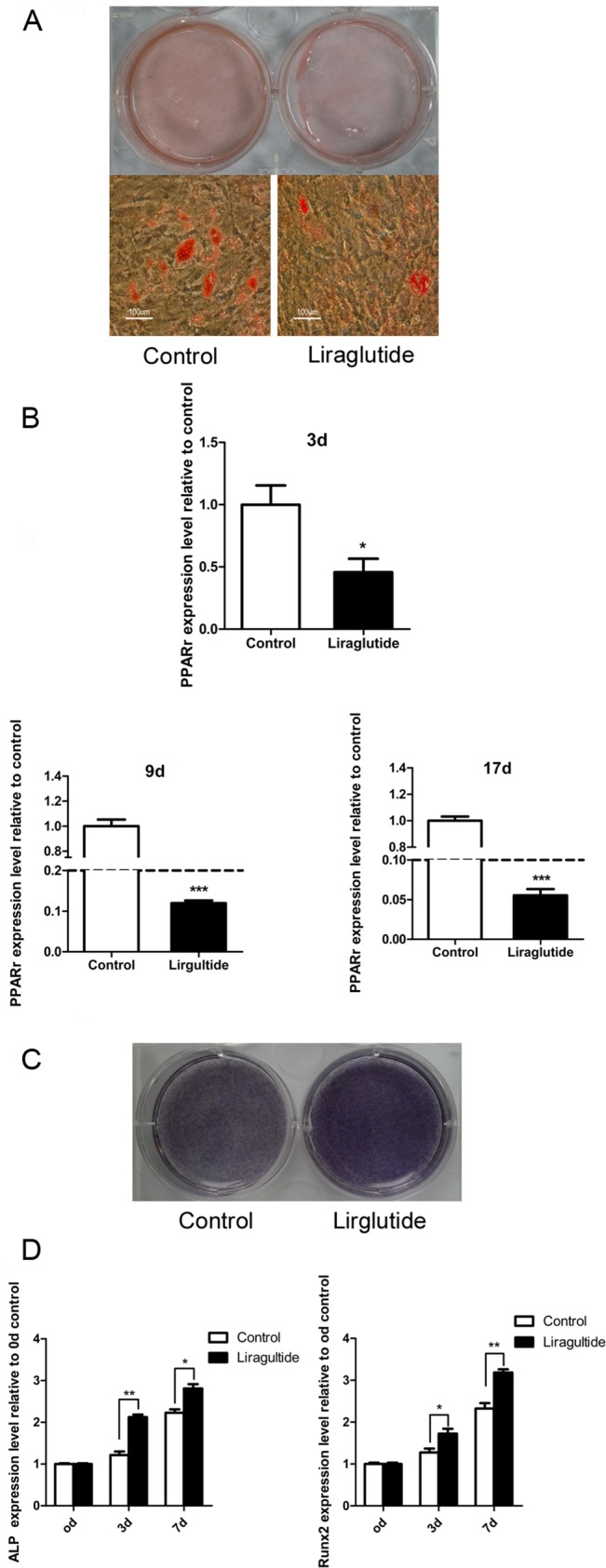
Effects of Liraglutide on adipogenesis and osteoblastogenesis of human BMSCs. Primary human BMSCs were induced to differentiate into adipocytes or osteoblasts in the presence or absence of 10 nM Liraglutide. (A) Oil red O adipocyte staining was performed after 17 days of adipogenesis induction. (B) Total RNA was extracted and subjected to real-time PCR. The graph shows the mRNA expression levels of PPARγ at the indicated time-points. (C) ALP staining was performed after 7 days of osteogenesis induction. (D) Real-time PCR analysis was conducted to compare the expression levels of osteoblastogenesis-related genes in the vehicle-treated culture and Liraglutide-treated culture at the indicated time-points during osteoblastogenesis differentiation. Values are expressed as the mean±SE.*, P<0.05; **, P<0.01; ***, P<0.001.

Finally, we examined whether Liraglutide exerted similar actions on human BMSCs. The attenuated Oil red O staining (Fig 5A) and decreased PPARγ expression (Fig 5B) suggested that Liraglutide also inhibited human BMSC adipogenesis. Moreover, in agreement with previous results observed in rat BMSCs, Liraglutide similarly enhanced osteoblastogenic processes in human BMSCs based on enhanced ALP staining (Fig 5C) and an increased expression pattern of Runx2 and ALP during the early stages of osteoblast differentiation (Fig 5D).

## Discussion

The major findings of the current study were that the anti-diabetic drug Liraglutide improved bone quality and increased BMD in osteoporotic OVX rats.

Recently, the potential beneficial role of novel anti-diabetic GLP-1RAs in skeleton metabolism in diabetic rodents and patients has become a hot topic [26–28]. Hyperglycemic conditions could compromise osteoblastogenesis and osteoclastogenesis and even impair embryonic skeleton development [29, 30]; thus, the anabolic bone effects of GLP-1RAs observed in diabetic models in preclinical and clinical studies may have been a secondary outcome of the improvement of glucose control. To address whether these effects could be replicated in non-diabetic osteoporotic rodents, we treated OVX rats without diabetes with Liraglutide. Liraglutide improved the trabecular volume, thickness and number, increased BMD, and reduced trabecular spacing in the femur in these osteoporotic rats. Similar effects were observed in the lumbar vertebrae of OVX rats treated with Liraglutide. These findings suggested that the osteogenic bone effects of GLP-1RAs could be applied to non-diabetic osteoporotic rodents. It was noteworthy that although Liraglutide treatment in OVX rats improved BMD and bone quality, it still could not restore these indices back to those of the SHAM rats. We also explored the possible bone effect of Liraglutide on non-diabetic-non-osteoporotic sham rats. As illustrated in S1 Fig, no significant enhancement of histomorphometric parameters was observed in the bone-sound animals after 8 weeks of treatment of Liraglutide. Further studies are needed to investigate whether the bone effects of Liraglutide are only observed in osteoporotic rodents, but not in normal rodents.

In the current study, we found that OVX rats without diabetes demonstrated improved glucose tolerance after treatment with Liraglutide. This finding echoes the results reported from a mouse study showing that 6 weeks of treatment with Liraglutide in normal glycemic mice resulted in increased insulin sensitivity and β cell function, accompanied by reduced β cell mass [31]. The 8.4% lower body weight in the Liraglutide-treated OVX rats compared with the vehicle-treated rats at the end of our study might be an additional factor responsible for this change. However, we hypothesize that the bone effects of Liraglutide occur independent of the blood glucose-lowering effect. Apparently, the poor bone quality displayed in OVX rats was a direct consequence of the ovariectomy. The reduction in bone quality was not caused by hyperglycemia because OVX rats and sham rats had similar fed state glucose and glucose tolerance levels in our study. Furthermore, human studies such as the Action to Control Cardiovascular Risk in Diabetes (ACCORD) trial, which was the first randomized trial to evaluate the effect of intensive glycemia therapy on fractures and falls, demonstrated that intensive glycemia control had a neutral effect on the occurrence of non-spine fractures, falls and BMD during the 3.8 years of follow-up [32]. Thus, improvement of glucose control does not necessarily relate to a better outcome for the skeleton. Accordingly, the bone effects of Liraglutide in non-diabetic rodents are not derived from its anti-diabetic efficacy.

Increasing evidence indicates that GLP-1 signaling can directly modulate skeleton homeostasis. Both glucose-dependent insulinotropic polypeptide (GIP) and GLP-1 receptors have been shown to be expressed on osteoblasts [11, 33, 34]. Mice without GLP-1 receptors have cortical osteopenia, reduced bone strength, and increased osteoclastic numbers and bone resorption activity. Moreover, the maturity of the collagen matrix is significantly reduced in these GLP-1-deficient mice [35, 36]. Double knock-out GIP-R and GLP-1R mutant mice showed less bone strength compared with age- and sex-matched WT mice [37]. All of these results point to the conclusion that the activation of the GLP-1 receptor may have direct osteogenic effects. Indeed, another GLP-1RA (exendin-4) was recently reported to improve bone strength and ameliorate bone micro-architecture by inhibiting bone resorption and enhancing bone formation in old OVX rats [15]. Thus, the anti-osteoporotic efficacy of GLP-1RAs in non-diabetic animals could be expected.

Little is known regarding the possible mechanism responsible for the favorable influence of GLP-1RA on the bone, even in diabetic rodents [27]. The multi-lineage fate of BMSCs is determined by several lineage-specific transcription factors (i.e., Runx2) that can stimulate the expression of other osteoblast-specific genes during osteoblastogenesis, including ALP, collagen α1, osteocalcin, and PPAR-γ. This effect represents a key molecular switch that can drive the differentiation of multipotent BMSCs towards adipocytes rather than osteoblasts [38, 39]. In this study, there was a significant reduction in PPAR-γ expression, a reduction in lipid accumulation, and an up-regulation in the expression of the osteoblast-specific transcriptional factor Runx2 and the osteoblast markers ALP and Col-1 during rat and human BMSC differentiation in the presence of Liraglutide. Similar results were also reported in an *in vitro* study in which human BMSCs were treated with GLP-1 [16].

Our findings clearly indicated that Liraglutide could facilitate osteoblastogenic differentiation and restrain adipogenic differentiation in both rat and human BMSCs. We did not corroborate the molecular mechanisms by which Liraglutide enhanced the expression of Runx2 and its target genes while inhibiting PPAR- γ expression in BMSCs, but previous research showed that the GLP-1 receptor was expressed on BMSCs and that the effects of GLP-1 on BMSCs were mediated through the MEK and PKC signaling pathways [16]. In the current study, we failed to observe enhanced alizarin deposition during osteoblastogenic differentiation following 21 days of Liraglutide treatment. Osteoblast responses to GLP-1 are dependent on their stage of differentiation. GLP-1 receptors are most abundantly expressed on less mature osteoblastic cells, but are barely expressed on most mature osteoblasts[40]. Because calcium deposition is performed by mature osteoblasts, it is likely that this marker will not significantly change upon Liraglutide treatment. In contrast, the expression level of Runx2, a critical transcriptional factor that facilitates the expression of other osteoblast-specific genes during the early stage of osteogenesis, was increased when BMSCs were treated with Liraglutide. These findings indicated that Liraglutide mainly targeted the early stage of osteoblast differentiation.

We did not explore the underlying mechanism responsible for the changes in BMDs in Liraglutide-treated OVX rats. In rodent models, Liraglutide can stimulate calcitonin release, which can bind to receptors on rat osteoclasts and inhibit bone resorption [35, 41]. The fact that we did not measure serum bone resorption markers and calcitonin levels is a limitation of the current study; thus, it is not clear whether the increased BMDs in Liraglutide-treated OVX rats were derived from the elevation of circulating calcitonin levels and suppressed osteoclastic activities. Another limitation of our study was that we did not quantify ALP activity. However, it was reported that ALP staining was also useful for displaying the osteoblastogenic differentiation level[24].

It is worthwhile to emphasize that although the nonglycemic use of GLP-1RAs in non-diabetic animal models or patients is promising, the results are not conclusive [42]^.^ Moreover, some side effects of GLP-1RAs have been described, such as pancreatitis, thyroid C cell hyperplasia or medullary thyroid cancer, and an increase in heart rate[8]. Large prospective randomized clinical trials and long-term follow up are required to confirm the bone efficacy and safety in both diabetic and non-diabetic patients [8].

Taken together, the current study clearly showed that the GLP-1RA Liraglutide is a skeletal anabolic agent in osteoporotic OVX rats. Its effects are possibly mediated by promoting osteoblastogenesis and restraining adipogenesis during BMSC differentiation. If these results are confirmed in large-scale human studies, treatment with Liraglutide will greatly benefit the vast majority of postmenopausal osteoporotic women.

## Supporting Information

S1 Fig Effects of Liraglutide on the bone micro-architecture of vertebra in SHAM rats. (A) Representative micro-CT images of the 5th lumbar vertebra detected by an Inveon CT scanner (Siemens, Germany) at a 50μm resolution. The figures present the coronal view, axial view, sagittal view and three-dimensional visualization of the trabecular micro-architecture in the different groups. (B) The trabecular micro-architecture parameters of the 5th lumbar vertebra were shown as: BV/TV, Tb.Th, Tb.N, and Tb.Sp. Eight-week-old female Wistar rats were subjected to a sham ovariectomy (SHAM, black bar); then, 3 months after surgery vehicle or Liraglutide (0.6mg/day; SHAM+L, white bar) were administered for 8 weeks. Values are expressed as the mean±SE; n = 6 rats per group.
(TIF)
