# Supplementary figures and images for: Glucagon-like peptide-1 receptor agonist Liraglutide has anabolic bone effects in ovariectomized rats without diabetes
Source: PLoS One. 2015 Jul 15;10(7):e0132744. doi: 10.1371/journal.pone.0132744 (PMC4503456; doi:10.1371/journal.pone.0132744)

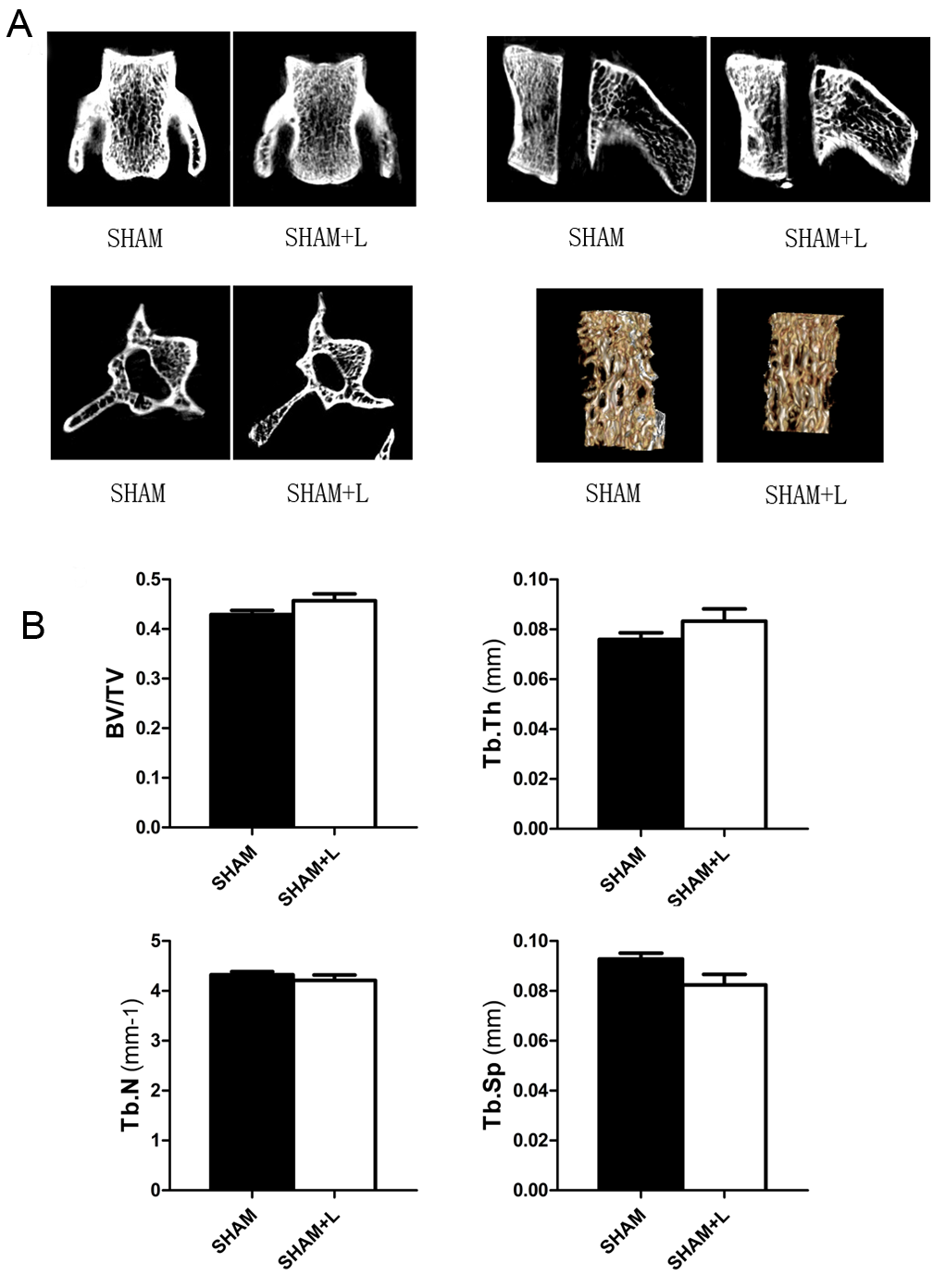

Supplement: S1 Fig — (A) Representative micro-CT images of the 5th lumbar vertebra detected by an Inveon CT scanner (Siemens, Germany) at a 50μm resolution. The figures present the coronal view, axial view, sagittal view and three-dimensional visualization of the trabecular micro-architecture in the different groups. (B) The trabecular micro-architecture parameters of the 5th lumbar vertebra were shown as: BV/TV, Tb.Th, Tb.N, and Tb.Sp. Eight-week-old female Wistar rats were subjected to a sham ovariectomy (SHAM, black bar); then, 3 months after surgery vehicle or Liraglutide (0.6mg/day; SHAM+L, white bar) were administered for 8 weeks. Values are expressed as the mean±SE; n = 6 rats per group. (TIF) [file pone.0132744.s001.tif]
